# Clinical Pharmacology of Bulevirtide: Focus on Known and Potential Drug–Drug Interactions

**DOI:** 10.3390/pharmaceutics17020250

**Published:** 2025-02-14

**Authors:** Martina Billi, Sara Soloperto, Stefano Bonora, Antonio D’Avolio, Amedeo De Nicolò

**Affiliations:** Department of Medical Sciences, University of Turin, Amedeo di Savoia Hospital, C.so Svizzera 164, 10149 Turin, Italy; martina.billi@unito.it (M.B.); stefano.bonora@unito.it (S.B.); antonio.davolio@unito.it (A.D.); amedeo.denicolo@unito.it (A.D.N.)

**Keywords:** bulevirtide (Hepcludex^®^, Myrcludex-B), hepatitis D virus, hepatitis B virus, pharmacokinetics, pharmacodynamics, drug–drug interactions

## Abstract

**Background:** Hepatitis D virus (HDV) is a defective virus requiring co-infection with hepatitis B virus (HBV) to replicate, occurring in 5% of HBV+ patients. Bulevirtide (BLV) is now the first-in-class specific anti-HDV agent, inhibiting HDV binding to NTCP, with good tolerability and good virological and biochemical response rates. Currently, little is known about its pharmacokinetic/pharmacodynamic (PK/PD), as well as potential drug-drug interaction (DDI) profile. In this work we provide a systematic review of the current knowledge on these aspects. **Methods:** A literature review of PK, PD and DDI profiles of BLV was conducted from Pubmed and EMA websites. Experimentally tested interactions and hypothetical mechanisms of interaction were evaluated, mostly focusing on usually co-administered anti-infective agents and other drugs interacting on NTCP. **Results:** BLV shows non-linear PK, due to target-mediated drug disposition, so its PK as well as PD is expected to be influenced by interactions of other drugs with NTCP, while it is not substrate of CYPs and ABC transporters. In-vivo investigated DDIs showed no clinically relevant interactions, but a weak inhibitory effect was suggested on CYP3A4 in a work when used at high doses (10 mg instead of 2 mg). In vitro, a weak inhibitory effect on OATP transporters was observed, but at much higher concentrations than the ones expected in vivo. **Conclusions:** The drug-drug interaction potential of BLV can be considered generally very low, particularly at the currently approved dose of 2 mg/day. Some attention should be paid to the coadministration of drugs with known binding and/or inhibition of NTCP.

## 1. Introduction

Nowadays, hepatitis B represents the major cause of acute and chronic liver disease in humans. In 2019, the WHO estimated that approximately 296 million people are affected by hepatitis B virus (HBV) infection worldwide, with about 5% displaying co-infection with delta virus (HDV), with major geographical hotspots represented by Mongolia, the Republic of Moldova, and countries in western and central Africa [1,2].

HBV infection leads, in most cases, to a chronic hepatitis (CHB), which yields progressive fibrosis up to liver cirrhosis and hepatocellular carcinoma. Among symptoms, fatigue, nausea/vomiting, abdominal pain, jaundice, and dark urine can be observed. In patients with cirrhosis, portal hypertension could cause esophageal varices, with high risk of hemorrhage, and many other symptoms associated with hepatic insufficiency, as hemostatic disorders, hypoalbuminemia, hyperammonemia, with consequential complications, such as ascites and neurological toxicity [3].

HDV is a defective human virus that requires HBV co-infection to replicate, since it lacks genetic information encoding envelope proteins, which is thus provided by HBV, which acts as a “helper virus” [4]. By a clinical point of view, HBV–HDV co-infection accelerates and worsen the natural history of CHB [5].

HDV is mainly transmitted by parenteral and sexual contact or vertically from infected mother to child, sharing the same transmission routes with HDV/HCV/HIV; this represents a major cause of co-infection [6].

Since the 1980s, the most recommended treatment for HBV and HDV infections has been Peg-IFNα-2a, which functions as immunomodulator inducing IFN-stimulated genes (binding Type I IFN receptor on hepatocytes), with the aim to obtain seroconversion, with the loss of HBsAg and the production of high titers of HBsAb [7]. Nevertheless, the most relevant contraindications of IFN α treatment are advanced portal hypertension, thrombocytopenia, or autoimmune disorders, precluding this type of treatment for a large proportion of HDV-infected people. On the other hand, a safer alternative anti-HBV and HDV treatment is Peg-IFN lambda, an immune modulator targeting Type III IFN receptors [8]. Unfortunately, while a high proportion of patients with HBV infection are currently treated with nucleoside retrotranscriptase inhibitors (NUCs), such as tenofovir (TFV, as disoproxil fumarate prodrug, TDF, or alafenamide, TAF) or entecavir (ETV), which present great effectiveness in reducing HBV DNA levels, these drugs are not capable of affecting HDV replication, since they do not inhibit the production of HBsAg, the structural protein lacking in HDV [8].

All the abovementioned treatment strategies, however, are not based on the inhibition of HDV-specific enzymes or receptors and, basically, affect HDV replication indirectly by the inhibition of the “helper virus”.

Recent attempts to obtain HDV-specific treatments are represented by the prenylation inhibitor lonafarnib (a repurposed drug, approved for the treatment of progeria syndrome) and the entry inhibitor bulevirtide (BLV) [8].

**Figure 1:**
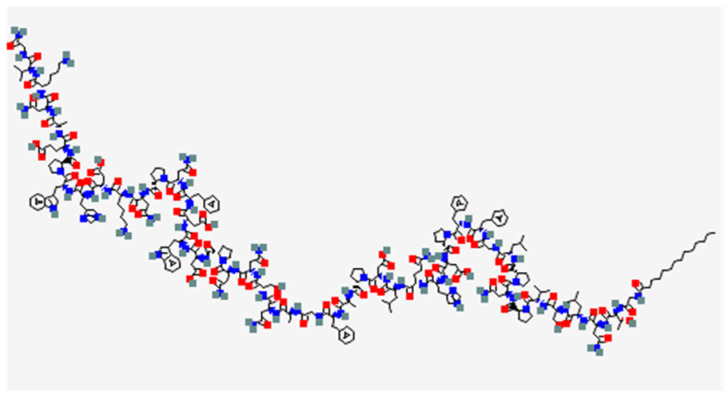
Bulevirtide chemical structure. Blue = Hydrogen, Red = Oxygen, Green = Nitrogen (Data from Pubchem [9]).

BLV (Hepcludex^®^, previously known as Myrcludex-B), whose chemical structure is showed in Figure 1, was developed by MYR GmbH for the treatment of chronic hepatitis delta (HDV) and chronic hepatitis B virus (HBV) infections. It consists in a myristoylated peptide capable to bind with high-affinity sodium taurocholate co-transporting peptide (NTCP), which works as a receptor for both HBV and HDV, inhibiting the viral entry within the hepatocytes.

This drug was first approved in July 2020 in the EU for HDV RNA-positive adult patients with compensated liver disease. It is administrated subcutaneously (SC injection) as a monotherapy or combined with a nucleoside/nucleotide analogue to treat the underlying HBV infection [10]. According to the EASL Clinical Practice Guidelines on hepatitis delta virus [11], BLV should be considered for administration in people with chronic hepatitis delta (CHD); dose and duration of treatment are not well defined yet, but long-term treatment with BLV, 2 mg once daily, may be considered. The combination with pegIFNα can be considered in patients who do not show specific contraindications for interferon.

Considering its recent introduction, little is known about BLV’s drug–drug interaction profile.

Given HBV/HDV/HCV/HIV co-infection is often possible [6,12], and since BLV is a new drug, possible drug–drug interactions (DDIs) between BLV and HIV/HCV-approved therapies could be present, but they have not been investigated yet.

In this context, this review aims to summarize the current available literature about BLV pharmacokinetic and pharmacodynamic properties, with particular focus on drug–drug interactions and to speculate on the potential DDIs still uninvestigated based on the current knowledge of BLV pharmacokinetic profile.

## 2. Data Sources

**Figure 2:**
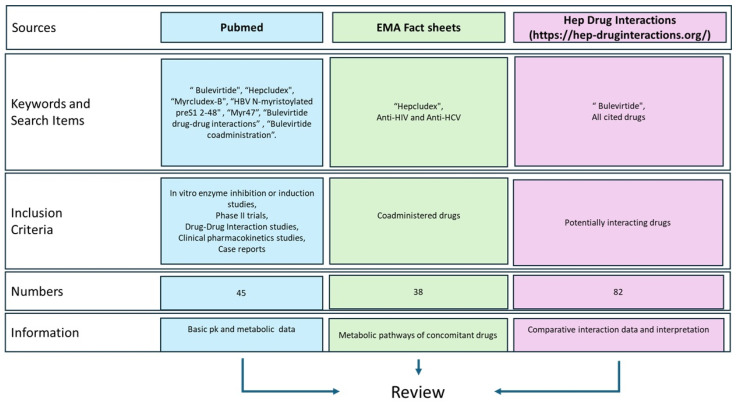
Data sources’ graphical representation. https://hep-druginteractions.org/ (accessed on 16 December 2024).

As summarized in Figure 2, this review was conducted by identifying literature from Pubmed, the European Medicines Agency (EMA) website, and the Hep Drug Interaction (University of Liverpool).

The pharmacokinetic and pharmacodynamic profiles of Bulevirtide were investigated using, as keywords: “Bulevirtide”, “Hepcludex”, “Myrcludex-B”, “HBV N-myristoylated preS1 2-48”, and “Myr47” on the websites previously cited. Subsequently, studies that investigated the interactions of BLV with other drugs were identified using keywords such as “Bulevirtide drug-drug interactions” or “Bulevirtide coadministration”. Finally, the EMA boards for anti-HIV and anti-HCV treatments were consulted to summarize the main molecular interactions of each drug [13].

## 3. Pharmacodynamic Properties, Efficacy, and Safety

**Figure 3:**
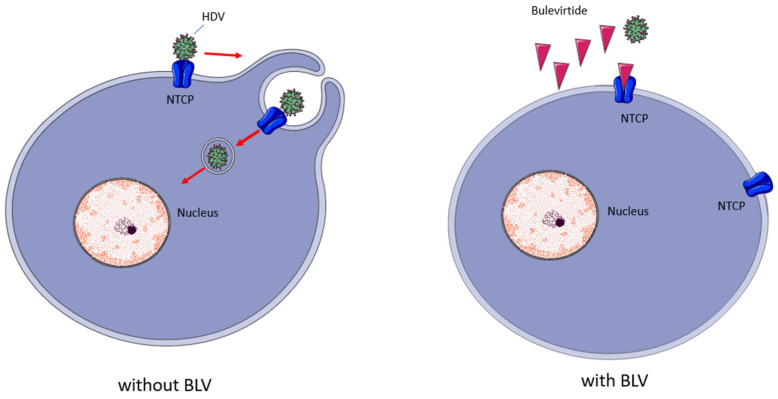
Bulevirtide’s mechanism of action. BLV binds with high affinity to NTCP, reducing the availability of this receptor to bind HDV, reducing the infection of new hepatocytes. Red arrows indicate steps in viral enrry; Red triangles indicate BLV molecules.

BLV is a myristoylated peptide containing 47 amino acids; it derives from the preS1-domain of the HBV L-surface protein and, as shown in Figure 3, its mechanism of action consists in inhibiting the virus entrance by binding to the hepatic NTCP (SLC10A1), a bile acids transporter which acts as a receptor for HBV and HDV entry into hepatocytes [14,15].

Nevertheless, while its effect as an entry inhibitor is expected both on HBV and HDV, in its clinical use, the net effect is particularly observed on HDV [16,17].

NTCP receptors are involved in the reuptake of bile acids from the portal circulation to the hepatocytes. Consistently with the physiological activity of NTCPs and the inhibitory activity exerted by BLV on NTCPs, an increase in plasma bile acids levels has been observed upon treatment of healthy volunteers [14,18]. In turn, this can lead to mild dermatological side effects (mainly pruritus) [18].

Phase I trials in humans testing BLV started in 2015 and have confirmed a good safety profile of BLV [19,20]. The following phase II and III studies (“MYR” trials) aimed to testing the safety and efficacy of BLV as monotherapy or co-administrated with tenofovir prodrugs and/or pegylated interferon α-2a (pegIFNα), which represents the only drug available for the treatment of hepatitis delta [16,17,20,21,22,23,24].

In an open-label phase 2b clinical trial, Wedemeyer and Bogomolov tested BLV 2, 5, and 10 mg daily doses combined with TDF vs. TDF alone for a short course of 24 weeks, showing end-of-treatment virological responses rates of 46.4%, 46.8%, and 76.6% vs. 3.3%, respectively, confirming the effectiveness of BLV and suggesting that higher doses could yield higher response [25].

Recently, the efficacy and tolerability of BLV has been demonstrated in several cohorts, alone and in combination with Peg-IFNα [26,27,28,29].

The results from the phase III study MYR301 showed a relatively satisfactory rate of virological response with a daily BLV dose between 2 and 10 mg, a general improvement in patients’ perceived quality of life, and a significant benefit from a long treatment course of 96 w against 48 w, with minimal differences between 2 and 10 mg/day at 96 w [22,30].

The work from De Gasperi et al. recently tested the current standard 2 mg qd BLV dose in patients with clinically significant portal hypertension, observing a 78% virological response, 83% biochemical response, and 67% of combined response after a treatment course of 48 weeks. Interestingly, a general improvement of hepatic markers and clinical conditions, without significant adverse events, was observed; only an asymptomatic increase of bile acids concentrations was observed, as expected [31].

Other works evaluated the performance of BLV alone or combined with pegylated interferon for chronic hepatitis D at high (10 mg/day) and low (2 mg/day) doses and against peg-IFNα alone. One trial showed superior performance in terms of SVR of the combination of 10 mg qd BLV with Peg-IFNα compared to Peg-IFN alone, low-dose BLV (2 mg qd) plus IFN alone, and high-dose (10 mg qd) BLV alone (46% vs. 25% vs. 26% vs. 12%, respectively) [23]. This synergistic effect was confirmed also by Wedemeyer et al. in a randomized trial, showing 86.7% vs. 40.0% SVR rates in favor of combined therapy after 48 weeks of treatment [32]. It is worth noting that, despite this higher effectiveness, the combination therapy is also associated with the common side effects of Peg-IFNα, which could reduce patients’ adherence to the therapeutic schedule [23].

General safety of BLV at a standard dose for at least 48 weeks with or without Peg-IFNα was also recently demonstrated in people living with HIV and HBV/HDV co-infection, without evidence of serious adverse events due to DDIs with anti-HIV drugs and with further evidence of increased effectiveness in the Peg-IFNα arm. Nevertheless, the rate of relapse of HDV-RNA after the end of treatment remained high in both arms [33].

Taking these data together, it is evident that BLV can yield a significant antiviral effect against HDV and strong beneficial effect in terms of biochemical response (e.g. ALT normalization), although better success rates can be reached by increasing the length of treatment, by combining it with Peg-IFNα, and/or by increasing its dose. These approaches are still subjects of investigation.

## 4. Bulevirtide Pharmacokinetics

A non-linear BLV pharmacokinetic profile was observed in healthy volunteers, following a two-compartment target-mediated drug disposition (TMDD) model [10,20]. Following SC administration, BLV bioavailability was estimated to be 85%.

According to TMDD, its exposure was found to increase disproportionally with an increasing dose following sub-cutaneous (SC) and intravenous administration, reducing the clearance and the apparent volume of distribution. This phenomenon is explained by the saturation of the receptor at higher doses, overcoming the capability of NTCP to cause TMDD and leading to higher concentrations in extrahepatic compartments, including plasma.

Steady-state BLV is expected to be reached within the first weeks under 2 mg regimen administration, with ~2-fold accumulation ratios for its maximum concentration (C_max_) and area under the curve (AUC). Time to C_max_ (T_max_) was found to be 1–3 h over a BLV dose range of 0.8–10 mg. Nevertheless, a more recent work showed a higher accumulation ratio later in treatment (at 85 days), up to nearly five-fold for both C_max_ and AUC [34]. This work showed C_max_ values slightly above 100 ng/mL (about 0.02 µM) at a daily dose of 5 mg, higher than the currently approved dose of 2 mg/day, remaining well below the concentrations described to be capable of inhibiting OATP transporters (see next section).

Even drug metabolism and elimination involve TMDD, since the main elimination mechanism depends by BLV binding to NTCP and the following degradation of this complex. This leads to a variable half-life in the range of 4–7 h.

## 5. In Vitro Enzymes and Transporters Inhibition Tests

In addition to interacting with the hepatic sodium taurocholate co-transporting polypeptide (NTCP), BLV was observed to be capable of inhibiting OATP1B1/3 transporters in vitro [10,18] at concentrations above 0.5 µM, of weakly inhibiting CYP1A2/2B3/2C9/2C19 at very high concentrations (10 µM) [35], and, in vivo, of weakly inhibiting CYP3A4 and CYP7A1 at a 10 mg daily dose [35]. No direct inductive activity on CYP450 isoenzymes or efflux transporters was observed in vitro, nor was direct binding to PXR receptor. These characteristics suggest an extremely low interaction potential as a perpetrator of pharmacokinetic drug–drug interactions, particularly at the current approved dose of 2 mg/day.

## 6. Drug–Drug Interactions

In this section, the theoretical drug–drug interactions of BLV are described, including both those as a victim and as a perpetrator. Patients with HDV infection are often affected by co-morbidities which need different pharmacological treatments (e.g., co-infection with HIV), thus increasing the need for a deep evaluation of the drug–drug interaction profile.

Due to the very recent introduction of BLV in clinical practice, the majority of these were not observed or confirmed experimentally in clinical trials.

### 6.1. Potential Drug–Drug Interactions Already Investigated in Humans

#### 6.1.1. Tenofovir

The co-infection with HIV in HBV infected patients has been demonstrated to worsen and accelerate the course of liver disease [36]. Additionally, among the 37.9 million people living with HIV in 2018, 5–20% are estimated to be co-infected with HBV, and about 5% of those infected with HBV are co-infected with hepatitis D virus [37].

Tenofovir (TFV), in the form of TDF and, more recently, TAF, is a nucleoside analogue commonly used for the treatment of HIV and HBV infections. TFV prodrugs are administered orally, with good bioavailability. Both the prodrugs are substrates of P-gP, but they are not substrates of CYPs. The main active circulating metabolite, TFV, is a substrate of organic anion transporters 1 and 3 (OAT1 and OAT3) [38,39].

BLV showed the capability to increase circulating levels of bile acids which, in turn, are weak inhibitors of organic anion transporter 3 (OAT3); this could lead to the reduced hepatic uptake and renal secretion of OAT3-specific substrates, such as TFV. Nevertheless, the recent study by Blank et al. demonstrated the absence of a clinically relevant PK interaction between BLV and TFV, suggesting that their concomitant administration is virologically effective and well tolerated [18].

#### 6.1.2. Pravastatin

Another study, conducted by Zhu et al. [14], assessed BLV interaction potential and PK with the OATP1B marker substrate pravastatin.

As a liver-specific uptake transporter, OATP1B contributes to the targeting of many substrate drugs from different classes and, most importantly, lipid-lowering statins, whose adverse events are closely linked to drug–drug interactions.

Pravastatin was proposed by the Food and Drug Administration (FDA) as a marker for drug interaction trials to evaluate OATP1B1 and OATP1B3; it does not undergo relevant CYP450 metabolism, and it is mainly excreted unchanged, prevalently (80%) by biliary excretion. These peculiar PK features make it a good marker of OATP activity [40,41].

The primary objective of Zhu et al. was to evaluate the effect of a 5 mg dose of BLV given twice a day (bid) and dosed to a steady state on the pharmacokinetics of a single dose of 40mg pravastatin. The results showed that the average exposure of pravastatin increased by only 32% with BLV at a steady state. Moreover, the pharmacokinetic profile of BLV was not influenced by the co-administration of pravastatin. This indicates a mild, and probably clinically irrelevant, capability of BLV to act as an inhibitor of OATP1B activity within the liver. Therefore, BLV shows some potential to act as a perpetrator, but not a victim, of OATP1B1-based PK interactions at high doses.

In the same study, other secondary objectives were the evaluation of the pharmacodynamic effects on plasma bile acid concentrations at steady-state BLV, which showed that the geometric mean AUC 0-12 of total bile acids increased 35.2-fold from 49.8 µmol/L·h at baseline to 1752 µmol/L·h (*p* < 0.001), unconjugated bile acids by 3.62-fold (*p* = 0.001), taurine-conjugated bile acids by 61.5-fold, and glycine-conjugated bile acids by 38.0 fold (both *p* < 0.001).

The effect of the co-administration of BLV and pravastatin on CYP3A4 activity was also assessed: a very small, clinically irrelevant 1.17 increase of estimated partial metabolic clearance (eClmet), derived from midazolam AUC2-4, was observed at BLV steady-state, suggesting that CYP3A4 substrates are not affected by BLV to a meaningful extent, even at a high dose. Table 1 summarizes the proven interactions described above.

### 6.2. Possible Interactions with Anti-HIV and Anti-HCV Drugs

Tenofovir is the only anti-HIV drug that has been examined in a dedicated drug–drug interactions study in combination with BLV.

In addition to interacting with the hepatic sodium taurocholate co-transporting polypeptide (NTCP), a study published by Blank et al. in 2018 denoted BLV as a possible inhibitor of CYP3A4 and CYP7A1 and an inhibitor of the organic anion transporting polypeptide 1B1 and 1B3 (OATP1B1 and OATP1B3) activity, other than being a weak inhibitor of CYP1A2/2B3/2C9/2C19 [35].

These characteristics could lead to weak interactions with some anti-HIV or anti-HCV drugs, which are substrates of these enzyme and transporters, although likely not clinically significant.

In this regard, a recent clinical trial of BLV in 38 people living with HIV (PLWH) showed some effectiveness (>50% virological response at the end of treatment, but a high relapse rate) and excellent safety, with no serious adverse events or HIV virological failures reported. The antiretroviral regimens in this study were prevalently based on three drugs (91.9%), including lamivudine (8.1%), TAF/emtricitabine (59.5%) and TDF/emtricitabine (32.4%), integrase inhibitors (62.2%), and non-nucleoside retrotranscriptase inhibitors (32.4%). No patient received protease inhibitor-based regimens, classically more prone to DDI potential [33].

In the next subsections, we report the DDI potential of BLV with anti-HIV and anti-HCV drugs, based on a mechanistic approach considering in vitro and in vivo interaction with enzymes and transporters and each drug’s pharmacokinetic/pharmacodynamic profile and tolerability.

#### 6.2.1. OATP1B1 and OATP1B3

A study published by Blank et al. in 2018 demonstrated that BLV inhibits organic anion transporting polypeptide 1B1 and 1B3 (OATP1B1 and OATP1B3) activity in a dose-dependent manner [35].

Both these peptides are influx transporters, meaning they are involved in the extraction of drugs from the portal circulation to the hepatocytes participating in the elimination of drugs by metabolism or biliary excretion [42].

Nevertheless, this inhibition was significant at BLV concentrations higher than 0.5 µM and 8.7 µM, for OATP1B1 and OATP1B3, respectively: a concentration of 0.5 µM may be reached as C_max_ at very high BLV doses (10 mg or more), while 8.7 µM is not expected to be reached even at a 20 mg/day dosage (10-fold higher than the current standard daily dose) [20,35].

Among HIV approved therapies, inhibitory activities against OATP drug transporters have been demonstrated for Fostemsavir (OATP1B1) and Cobicistat (OATP1B1 and OATP1B3), so that the concomitant administration of BLV may show an additive or synergistic effect in this direction, increasing the DDI potential for their substrates [43].

On the other hand, the CCR5 inhibitor Maraviroc is a proven OATP1B1 substrate; the transporter mediates the uptake of the drug, affecting its pharmacokinetic profile [44].

Among HCV-antivirals, two NS5A replication complex inhibitors, Pibrentasvir and Velpatasvir, as well as the NS3/4A protease inhibitors Voxilaprevir, Paritaprevir, and Glecaprevir, have demonstrated inhibitory activity against OATP1B1 and 1B3, potentially increasing the DDI potential mediated by these transporters in the case of co-administration with BLV [45,46,47].

Based on EMA product information of HIV and HCV antivirals, with BLV being a very weak OATP1B1 and OATP1B3 inhibitor, it could be theoretically able, at a very high dose/concentration, to affect the metabolism of the substrates of these transporters. Among HIV and HCV drugs, possible victims are Tenofovir alafenamide (TAF, anti-HIV), Grazoprevir, Paritaprevir, and Glecaprevir (anti-HCV protease inhibitors). Nevertheless, this inhibition is extremely unlikely to be clinically relevant, considering the weak inhibitory effect exerted at high doses and the very high tolerability of these potentially interacting drugs.

#### 6.2.2. Cytochrome P450: CYP3A4/2C9/2C19

In vitro evaluation showed that very high concentrations (>10 µM) of BLV could inhibit all CYPs in a weak manner [35]. Nevertheless, these concentrations are never expected to be reached in vivo, since the highest reported concentrations, even at very high dose (20 mg, 10-fold higher than the current approved 2 mg/day dose), are consistently lower than 2 µM [18]. A recent work showed that some weak inhibition of CYP3A4 was observed in vivo in a cohort of patients co-treated with tenofovir disoproxil fumarate during treatment; nevertheless, this inhibitory effect was hypothesized as an indirect effect of the increased bile acids concentrations and was extremely weak, unlikely to be clinically significant [18].

Anyway, this evidence led to the recommendation to keep close monitoring on concomitant drugs (for instance, by therapeutic drug monitoring) with a very narrow therapeutic index, which are CYP3A substrates (e.g. cyclosporine, carbamazepine, simvastatin, sirolimus, and tacrolimus) [48].

A recent case report from Pinchera et al. (2024) [49] suggested a slight increase in the tacrolimus exposure despite dose reduction after the addition of BLV in a patient who received a kidney transplantation; nevertheless, it is worth noting that the observed variability in tacrolimus concentrations in the latter report was not higher than the one observed in previous reports [50]. Therefore, this evidence needs to be confirmed in wider courts or in randomized prospective studies.

As shown in Table 2, among the anti-HIV drugs, non-nucleosides antiretrovirals (Doravirine, Etravirine, Nevirapine, and Rilpivirine), many protease inhibitors (PIs, such as Atazanavir, Darunavir, Fosamprenavir, Ritonavir, Tipranavir, and Lopinavir), the CCR5 antagonist Maraviroc, integrase strand transfer inhibitors (Dolutegravir, Bictegravir, and Elvitegravir), the attachment inhibitor Fostemsavir, the capsid inhibitor Lenacapavir, and the pharmacokinetic enhancer Cobicistat are either metabolic substrates or inhibitors of CYP3A, showing opposite activity on the cytochrome compared with BLV and possibly affecting their metabolism.

Considering the anti-HCV drugs (Table 3), the NS5A replication complex inhibitors Pibrentasvir and Velpatasvir; the NS5B polymerase inhibitor Dasabuvir; and the NS3/4A protease inhibitors Voxilaprevir, Grazoprevir, Paritaprevir, and Glecaprevir are possibly similarly affected by the co-administration of BLV.

BLV is also a weak inhibitor of CYP1A2/2B3/2C9/2C19 at very high concentrations (10 µM); in particular, CYP2C9 and CYP2C19 are of major interest as for possible interactions [35]. Nevertheless, these concentrations are very higher from those expected in real-life use.

The following tables (Table 2 and Table 3) summarize the hypothesized interactions of BLV with anti-HIV and anti-HCV antivirals, respectively, classified according to the mechanism of action, as shown above.

### 6.3. Interactions with Other Drugs

Considering the peculiar PK/PD properties of BLV, and particularly the phenomenon of TMDD, the pharmacological effect is not only based on the binding of the drug to NTCP, but its distribution and clearance are also dependent on the binding.

In the recent years, NTCP has deserved attention as a possible druggable target for different hepatic disorders, and several drugs have shown some degree of binding and the capability to exert inhibitory activity [93]. Some of these drugs could even exert some minor anti-HDV activity, particularly in vitro [89].

Nevertheless, sharing the same molecular target with BLV could lead to different interaction scenarios, based on the binding site and the binding affinity. In detail, drugs which share the same binding site, have high affinity to the receptor, and/or high concentration could compete for BLV binding to NTCP, potentially reducing the net activity against the virus or showing an additive effect; drugs which bind on allosteric sites could change (positively or negatively) BLV binding to NTCP, with difficult to predict virological implications; drugs which bind the same NTCP site with low affinity could not significantly impact BLV PD, but could slightly decrease its clearance and its apparent volume of distribution, due to a reduced TMDD, thus increasing BLV exposure. Moreover, the addition of a concomitant drug with an inhibitory effect on NTCP could also show additional effect on the concentrations of bile acids, possibly increasing the risk for systemic toxicity. In Table 4, we report the main list of drugs which show inhibitory activity on NTCP. Beyond the above discussed interaction profiles with antinfective drugs, a list of hypothetical interactions potentials with other drugs has been provided in supplementary materials (Supplementary Table S1).

## 7. Discussion and Conclusions

BLV, also known as Hepcludex^®^, is a novel antiviral drug that has shown promising results in the treatment of chronic HDV infection. It works by inhibiting the entry of HDV into liver cells, thereby stopping the spread of the virus and reducing liver damage. This drug has the potential to revolutionize the treatment of HDV, a chronic and severe form of viral hepatitis that affects millions of people worldwide.

Bulevirtide is a synthetic peptide derived from the preS1 domain of the hepatitis B virus (HBV) surface antigen. It acts as an entry inhibitor by binding to the sodium taurocholate cotransporting polypeptide (NTCP) receptor on liver cells, thereby preventing the entry of HDV into hepatocytes. This mechanism of action is specific to bulevirtide and distinguishes it from other antiviral drugs used in the treatment of chronic hepatitis B and C. One of the potential problems for new therapies may be related to drug interactions that could alter both the pharmacokinetics and pharmacodynamics of the drugs involved. It is therefore important to know the interaction profile of the drug administered; therefore, in this review, the DDI potential of BLV has been reviewed.

A study by Blank et al. (2018) investigated the pharmacokinetic interactions between BLV and TDF, a commonly used antiretroviral drug for the treatment of hepatitis B. The results showed that BLV did not significantly affect the plasma concentrations of tenofovir, suggesting that there is no clinically significant drug–drug interaction between the two medications [18].

BLV does not appear to be a substrate of cytochrome P450, UDP-glucuronyl transferase isoforms or drug transporters, such as P-gP, and therefore its potential for DDIs as a victim for these “canonical” pathways is very low. Nevertheless, considering its recent approval [10], the literature is very scarce regarding its real-life use. What we know comes mainly from the preclinical studies carried out by the pharmaceutical company that marketed the drug. Its interaction potential as a perpetrator appears to be weak and may depend on its weak inhibitory effect on OATP1B1 and OATP1B3 transporters. Nevertheless, all the in vitro data suggest that this weak inhibitory activity is exerted at a very high concentration (0.5 µM, about 2700 ng/mL), considerably higher than the expected C_max_ in plasma at the steady state at any currently tested dosing, let alone the currently approved 2 mg/day dosing schedule, so it is very unlikely that this mechanism could cause clinically relevant DDIs, unless very higher dosing schedule is adopted. Nevertheless, considering the slight increase in pravastatin exposure in clinical DDI studies suggests that, in vivo, some effect on OATP1B1 and OATP1B3 transporters could be exerted at lower concentrations, probably through indirect effect of increased bile acids concentrations in plasma.

Some minor effects were observed as CYP inhibitor at very high concentrations in vitro (>10 µM), as well as a minor effect as a weak CYP3A4 inhibitor in vivo at a high dosage (10 mg/day): however, this effect is very unlikely to be clinically significant in most cases, both because it is weak and because of the current much lower approved dosage of 2 mg/day [35].

In fact, the only mechanism of interaction which presents some concern, since it could have some significant impact on the PK/PD of BLV in an unpredictable manner, is the one related to the co-administration of other inhibitors of NCTP. The function of NTCP as a transporter for bile acids is already known to be strongly affected by the treatment with BLV, so that a significant increase in their levels in plasma is the main collateral effect. Nevertheless, NTCP is known to bind some other drugs, including sulfasalazine, rosiglitazone, troglitazone, irbesartan, ezetimibe, cyclosporine A, and ritonavir, so that the effect of their co-administration on the PK and PD of BLV is difficult to predict [68,93,97,103,104,105]. This leads to a precautionary contraindication for the co-administration of these drugs with BLV. On the other hand, nothing is known about the in vivo impact of these possible DDIs, which may result in an increase in the BLV plasma exposure, due to lower TMDD, as well as an increased, unchanged, or even reduced PD effect, based on whether the inhibitory effect of concomitant drugs on NTCP results is additive or if their binding exerts any sort of competition with the one of BLV.

Moreover, it is important to note that some other antiretroviral drugs, such as atazanavir and lopinavir, as well as cobicistat, share a similar structure with ritonavir and have a possible binding and inhibitory effect NTCP, so the co-administration of these drugs should be taken with caution or be avoided.

In fact, these issues led to precautionary contraindications from the online interaction checker on the Liverpool Website [63,106,107]. It is worth noting that all these cases of contraindication are only putative and precautionary, based on very low-quality evidence and in the absence of DDI clinical trials or evidence from real-life clinical use.

Therefore, all these unclear aspects absolutely deserve further study, particularly concerning the interaction with boosted antiretroviral regimens, since the co-infection between HIV and HBV/HDV is relatively common. Waiting for high-quality evidence from clinical trials, these interactions could be investigated experimentally in vitro and by physiologically based PK (PBPK) modeling to obtain an approximate prediction of the PK effect.

Anyway, excluding currently unpredictable DDI with NTCP inhibitors, no clinically relevant interaction is present between BLV and anti-HIV and anti-HCV drugs, confirmed by a recent work testing BLV with or without Peg-IFNα in PLWH, which showed excellent tolerability.

Even considering the DDI potential with other drug classes, other than HIV drugs, this remains extremely contained, as we can see from Table 4. Nevertheless, considering some weak inhibitory effect on CYP3A4 at a very high dose was observed, some caution should be paid if co-administered with drugs with very narrow therapeutic ranges, such as immunosuppressants. This is completely expected, considering its peptide structure, its subcutaneous route of administration, and its peculiar metabolism based on TMDD, reflecting its non-linear PK. Therefore, its activity and possible rare concentration-related side effects should be mainly related to the patient’s physiology and much less to drug interactions that might be observed when co-administered with other molecules for other treatments. Among the most common side effects, there are appreciable increasing in bile acids, subsequent rash (pruritus), and probable metabolic problems.

However, it remains important what the pharmacological exposure is in each patient, and although interactions may be considered secondary factors, testing blood concentrations of this drug (where available) may remain an important strategy for predicting its efficacy/failure or the onset of toxic effects. For this aspect, however, data are still insufficient.

In conclusion, BLV is a promising antiviral drug for the treatment of chronic hepatitis D virus infection, with a very low DDI potential, both as a victim and as a perpetrator, particularly at the currently approved dosage of 2 mg/day. Its peculiar mechanism of action, favorable safety profile, and potential for achieving virological response and biochemical response make it a valuable addition to the existing treatment options for HBV/HDV.

Given that the optimal length of BLV therapy for achieving a lasting virological response is not yet determined, continuing BLV treatment for more than one year currently seems to be the most suitable approach to enhance or sustain the virological response as suggested by EASL [11].

Further research is needed to optimize the long-term efficacy and safety of BLV, particularly regarding the ideal dosing schedule, the length of the treatment course and the concomitant use of Peg-IFNα, as well as its unexplored potential DDIs with drugs which bind NTCP, in clinical practice. Healthcare providers should be aware of the pharmacological properties of BLV, which shows a very low (but not absent) interaction potential, when prescribing this medication to patients with chronic hepatitis D.

## Figures and Tables

**Table 1:** BLV drug–drug interaction currently investigated in-vivo.

Reference	Design	Population	Regimen	Pk Results	Clinical Implications
Zhu et al., 2023 [14]	Single-center, open-label, fixed-sequence drug–drug interaction trial	*n* = 19 healthy volunteers	5 mg dose of BLV, given twice a day (bid) + 40 mg single-dose 40 mg pravastatin	- The average exposure of the sensitive OATP1B marker substrate pravastatin increased by only 32% with BLV at steady state. - BLV pharmacokinetics were not influenced by the co-administration of pravastatin. - Genetically reduced OATP1B1 activity was associated with statin-induced myotoxicity in patients with *5 or *15 haplotypes. Our SCLO1B1*5/*15 and *15/*15 carriers had 11–28% lower pravastatin AUC values during BLV, which was in contrast to our findings in the wild-type group (with the strongest increase in pravastatin exposure in the homozygous wild-type group).	- Mild, but probably clinically unrelevant in vivo inhibition of OATP1B-mediated pravastatin uptake into the liver
Blank et al., 2018 [18]	Single-center, open-label, prospective trial assessing the influence of myrcludex B on steady-state tenofovir pharmacokinetics	*n* = 12 healthy volunteers	After basement assessment (trial day 1), days 2–7 245 mg of oral tenofovir disoproxil fumarate administrated once daily. Days 8–13: co-administration of 245 mg tenofovir + 10 mg myrcludex B subcuteneously (2 consecutive injections).	- Neither tenofovir AUC nor Cmax were significantly altered by myrcludex B. Myrcludex B had no significant effect on renal tenofovir clearance. - The co-administration of tenofovir and myrcludex B increased the mean overall plasma exposure of total bile acids. Moreover, renal excretion of bile acids increased almost 8-fold. - Compared to tenofovir monotherapy, the co-administration of tenofovir and myrculex B showed a weak inhibition of CYP3A activity (1.35-fold increase in midazolam AUC), expected to be not clinically relevant.	- No difference was observed in the pharmacokinetic profile between tenofovir monotherapy and tenofovir and myrcludex B co-administration, suggesting that the co-administration of the two drugs is safe and well tolerated. - The increase in total bile acids under myrcludex B treatment requires long-term monitoring of patients, although no serious adverse events were observed during the trial. - A weak influence of myrcludex B on CYP3A activity cannot neither be confirmed nor denied.

**Table 2:** Hypothesized interactions of BLV with anti-HIV antivirals, classified according to the mechanism of action.

Antiretrovirals NRTIs						
Drug	Metabolic Substrate of	Transport	Inducer of	Inhibitor of	Notes (Interaction with BLV)	References
Tenofovir disoproxil fumarate	Esterase enzymes in the gut and plasma			Weak inhibitor of cellular polymerases α, β, and γ	Low potential for interaction. In vivo data showed no interaction.	[51]
Emtricitabine				Weak inhibitor of cellular polymerases	Low potential for interaction. No evidences of common metabolic patways.	[52]
Lamivudine	Sulfotranferases			Weak inhibitor of cellular polymerases α, β	No evidences of common metabolic patways.	[53]
Tenofovir Alafenamide	Cathepsin A in vitro substrate of OATP1B1 and OATP1B3	P-gp; breast cancer resistance protein (BCRP)		Weak inhibitor of cellular polymerases α, β, and γ	BLV showed a weak inhibitory activity on OATP1B1 and OATP1B3, possibly affecting TAF metabolism. This inhibition was observed at the high 10 mg bid dose.	[54]
Abacavir	Alcohol dehydrogenase and glucuronyl transferase			Abacavir can potentially inhibit cytochrome P450 1A1 (CYP1A1). The drug shows a limited potential to inhibit CYP3A4-mediated metabolism.	Low potential for interaction.	[55]
Zidovudine	UGT enzymes				Low potential for interaction. No evidence of common metabolic pathways.	[56]
**Antiretrovirals NNRTIs**						
**Drug**	**Metabolic substrate of**	**Transport**	**Inducer of**	**Inhibitor of**	**Notes (interaction with BLV)**	**References**
Doravirine	CYP3A4		Weak inducer of CYP3A		A slight increase in doravirine exposure is possible with unlikely clinical relevance.	[57]
Efavirenz	CYP450		In vivo inducer of CYP3A4, CYP2B6 e UGT1A1. Possible inducer of CYP2C19 and CYP2C9.		It can induce possible late-onset neurotoxicity at concentrations above 6000 ng/mL (C trough). Clinically significant interaction is unlikely, but therapeutic drug monitoring of Efavirenz would be beneficial to improve the safety.	[58]
Etravirine	Etravirine is metabolised by CYP3A4, CYP2C9 and CYP2C19.		Weak inducer of CYP3A4	Weak inhibitor of CYP2C9, CYP2C19, and P-glycoprotein	No clinically relevant interaction is expected, considering the tolerability of Etravirine and the opposite effects on CYP3A4.	[59]
Nevirapine	CYP450, CYP3A		Inducer of CYP3A isoenzymes and potentially CYP2B6		No clinically relevant interaction is expected, considering the good tolerability of Nevirapine.	[60]
Rilpivirine	CYP3A			In vitro inhibitor of the MATE-2K transporter	No clinically relevant interaction is expected.	[61]
**Antiretrovirals PIs**						
**Drug**	**Metabolic substrate of**	**Transport**	**Inducer of**	**Inhibitor of**	**Notes (interaction with BLV)**	**References**
Atazanavir	CYP3A4			CYP3A4 Possible inhibitor of NTCP	Co-administration with NTCP inhibitors is not recommended as it can alter BLV elimination. Co-administration should be avoided.	[62,63,64]
Darunavir	CYP3A			CYP3A, CYP2D6 and P-gp	No clinically relevant interaction is expected considering the good tolerability of Darunavir.	[65]
Fosanprenavir	CYP3A4				No clinically relevant interaction is expected.	[66]
Ritonavir	CYP3A4, CYP2D6			CYP3A4 NTCP	Co-administration with NTCP inhibitors is not recommended as it may alter BLV elimination and/or its effect. Co-administration should be avoided.	[67,68]
Tipranavir	CYP3A P-gp (in vitro)		CYP3A	CYP3A CYP 1A2, CYP 2C9, CYP 2C19 and CYP 2D6 P-gp (in vitro)	No clinically relevant interaction is expected due to the good tolerability of Tipranavir.	[69]
Lopinavir	CYP3A			CYP450	Co-administration with NTCP inhibitors is not recommended as it may alter BLV elimination and/or its effect. Co-administration should be avoided.	[64,70]
**Antiretrovirals Fusion inhibitors**						
**Drug**	**Metabolic substrate of**	**Transport**	**Inducer of**	**Inhibitor of**	**Notes (interaction with BLV)**	**References**
Enfuvirtide					No evidence of common metabolic pathways.	[71]
**Antiretrovirals CCR5 antagonist**						
**Drug**	**Metabolic substrate of**	**Transport**	**Inducer of**	**Inhibitor of**	**Notes (interaction with BLV)**	**References**
Maraviroc	CYP3A4 and CYP3A5	Substrate of the transporters glycoprotein-P and OATP1B1			No clinically relevant interaction is expected.	[44,72]
**Antiretrovirals Integrase strand transfer inhibitors**						
**Drug**	**Metabolic substrate of**	**Transport**	**Inducer of**	**Inhibitor of**	**Notes (interaction with BLV)**	**References**
Cabotegravir	UGT1A1 UGT1A9 P-gp BCRP			OAT1 OAT3	No evidence of common metabolic pathways.	[73,74]
Dolutegravir	UGT1A1 UGT1A3 UGT1A9 CYP 3A4 P-gp BCRP			OCT2 MATE OAT1 OAT3	No evidence of common metabolic pathways.	[74,75,76]
Raltegravir	UGT1A1				No evidence of common metabolic pathways.	[74,77]
Bictegravir	CYP3A UGT1A1 Pgp BRCP				No clinically relevant interaction is expected due to high tolerability of bictegravir, its partial dependence on CYP3A4 and the weak inhibitory effect of BLV.	[74,78]
Elvitegravir	CYP3A UGT1A1/3		CYP2C9 UGT		No clinically relevant interaction is expected due to high tolerability of elvitegravir.	[74,79]
**Antiretrovirals post attachment inhibitors**						
**Drug**	**Metabolic substrate of**	**Transport**	**Inducer of**	**Inhibitor of**	**Notes (interaction with BLV)**	**References**
Ibalizumab	Metabolism studies not cunducted				There is no evidence of common metabolic pathways between Ibalizumab and BLV.	[80]
**Antiretrovirals capsid inhibitors**						
**Drug**	**Metabolic substrate of**	**Transport**	**Inducer of**	**Inhibitor of**	**Notes (interaction with BLV)**	**References**
Lenacapavir	CYP3A P-gp UGT1A1			CYP3A P-gp BCRP	Precautionary Lenacapavir TDM may be useful.	[81]
**Antiretrovirals pharmacokinetic enhancers**						
**Drug**	**Metabolic substrate of**	**Transport**	**Inducer of**	**Inhibitor of**	**Notes (interaction with BLV)**	**References**
Cobicistat	CYP3A CYP2D6			CYP3A CYP2D6 P-gp BCRP MATE1 OATP1B1 OATP1B3	No clinically relevant interaction is expected.	[43,82]
**Antiretrovirals attachment inhibitor**						
**Drug**	**Metabolic substrate of**	**Transport**	**Inducer of**	**Inhibitor of**	**Notes (interaction with BLV)**	**References**
Fostemsavir	P-gp BCRP CYP3A4 CYT P450			OATP1B1, BCRP, MATE	No clinically relevant interaction is expected.	[43,83]

**Table 3:** Hypothesized interactions of BLV with anti-HCV antivirals, classified according to the mechanism of action.

NS5A Replication Complex Inhibitor						
Drug	Metabolic Substrate of	Transport	Inducer of	Inhibitor of	Notes (Interaction with BLV)	References
Elbasivir	CYP3A P-gp			BCRP	No clinically significant interaction is expected.	[84]
Pibrentasvir/Glecaprevir	P-gp CYP3A			P-gp BCRP OATP1B1 OATP1B3 CYP3A UGT1A1 BSEP (bile salt export pump)	No clinically significant interaction is expected.	[45,85]
Ledipasvir	P-gp BCRP			P-gp BCRP	No clinically significant interaction is expected.	[86]
Velpatasvir	CYP2B6, CYP2C8, CYP3A4	P-gp BRCP OATP1B			As a precautionary measure, close clinical monitoring is warrented for coadministered narrow therapeutic index drugs which are sensitive to CYP3A4 and OATPB1/3 substrates. Co-administration of these substrates should be avoided.	[46,87]
Ombitasvir	Amide hydrolysis followed by oxidative metabolism			**UGT1A1**	No clinically significant interaction is expected.	[88]
**NS5B polymerase inhibitor**						
**Drug**	**Metabolic substrate of**	**Transport**	**Inducer of**	**Inhibitor of**	**Notes (interaction with BLV)**	**References**
Sofosbuvir	P-gp BCRP				No clinically significant interaction is expected.	[89]
Dasabuvir	P-gp BCRP OCT1 CYP2C8 CYP3A			**P-gp** **UGT1A1** **UGT1A4** **UGT1A6** **UGT2B7**	Precautionary Dasabuvir TDM may be useful.	[90,91]
**NS3/4A protease inhibitor**						
**Drug**	**Metabolic substrate of**	**Transport**	**Inducer of**	**Inhibitor of**	**Notes (interaction with BLV)**	**References**
Voxilaprevir	CYP3A4	P-gp BCRP		**P-gp** **BCRP** **OATP1B1** **OATP1B3**	Precautionary Voxilaprevir TDM may be useful.	[92]
Grazoprevir	OATP1B P-gp CYP3A BCPR (potectially)			BCRP	Precautionary Grazoprevir TDM may be useful.	[84]
Paritaprevir	CYP3A4 CYP3A5 OATP1B1 P-gp BRCP			OATP1B1 OATP1B3 P-gp (in vitro) UGT1A1	Precautionary Paritaprevir TDM may be useful.	[47,88]
Glecaprevir	P-gp CYP3A OATP1B1 OATP1B3			P-gp BCRP OATP1B1 OATP1B3 CYP3A UGT1A1 BSEP (bile salt export pump)	Precautionary Glecaprevir TDM may be useful.	[45,47,85]

**Table 4:** Hypothesized BLV interactions with other categories of drugs.

Drug Class	Drug	NTCP Interactions	Therapeutic Alternatives	References
Antivirals anti-HIV (PIs)	Atazanavir (Reyataz)	Possible inhibitor of NTCP	Other unboosted antiretroviral regimens, avoiding boosted PIs	[62,63]
	Ritonavir	NTCP inhibitor	-	[67,68]
Antifungals	Ketoconazole	NTCP inhibitor	Triazoles for systemic treatment	[94]
Antivirals	Brincidofovir	Metabolic substrate of NTCP		[95]
Treatment of hypothyroidism	Levothyroxine	Possible metabolic substrate of NTCP (since it is a thyroid hormone)	-	[96]
Lipid Lowering Agents	Ezetimibe	NTCP inhibitor		[97]
	Atorvastatin	NTCP inhibitor		[98]
	Fluvastatin	NTCP inhibitor		[99]
	Pitavastatin	NTCP inhibitor		[100]
	Pravastatin	NTCP inhibitor		[101]
	Rosuvastatin	NTCP inhibitor		[102]
Immunosuppressants	Cyclosporin	Metabolic substrate of NTCP	Tacrolimus, Sirolimus, and/or Everolimus	[103]
Hypertension, Heart Failure agents	Irbesartan	Metabolic substrate of NTCP	ARBs or ACE inhibitors	[104]
Gastrointestinal Agents	Sulfasalazine	NTCP inhibitor	Mesalazine	[105]

## Data Availability

No experimental data were generated from this study.

## Supplementary Materials

The following supporting information can be downloaded at: https://www.mdpi.com/article/10.3390/pharmaceutics17020250/s1, Table S1: Hypothesised BLV interactions with compound belonging to other drug classes. The information reported in this table was obtained from the *HEP Drug Interactions database* (University of Liverpool https://hep-druginteractions.org/checker, accessed on 16 December 2024).
