# Supplementary material for: Clinical Pharmacology of Bulevirtide: Focus on Known and Potential Drug–Drug Interactions
Source: Pharmaceutics. 2025 Feb 14;17(2):250. doi: 10.3390/pharmaceutics17020250 (PMC11859527; doi:10.3390/pharmaceutics17020250)
Supplement: Supplementary file 1 [file pharmaceutics-17-00250-s001.zip › pharmaceutics-3452922-supplementary.pdf]

pharmaceutics

Supplementary Materials: Clinical Pharmacology of Bulevirtide: Focus on Known and Potential Drug–Drug Interactions

Martina Billi, Sara Soloperto, Stefano Bonora, Antonio D'Avolio and Amedeo De Nicolò

Table S1. Other drug classes.

Drug	Metabolic substrate of	Inducer of	Inhibitor of	Notes (interaction with BLV)
Alfentanil (Analgesic)	CYP3A4			Weak inhibition of CYP3A4 by high dose (10 mg) BLV was observed in a study, unlikely to be clinically relevant particularly at standard 2 mg dose. Precautionary clinical monitoring is suggested.
Fentanyl (Analgesic)	CYP3A4			Weak inhibition of CYP3A4 by high dose (10 mg) BLV was observed in a study, unlikely to be clinically relevant particularly at standard 2 mg dose. Precautionary clinical monitoring is suggested.
Hydrocodone (Analgesic)	CYP3A4<br>CYP2D6			Weak inhibition of CYP3A4 by high dose (10 mg) BLV was observed in a study, unlikely to be clinically relevant particularly at standard 2 mg dose. Precautionary clinical monitoring is suggested.
Oxycodone (Analgesic)	CYP3A<br>CYP2D6			Weak inhibition of CYP3A4 by high dose (10 mg) BLV was observed in a study, unlikely to be clinically relevant particularly at standard 2 mg dose. Precautionary clinical monitoring is suggested.
Amiodarone (Antiarrhytmic)	CYP3A4<br>CYP2C8			Weak inhibition of CYP3A4 by high dose (10 mg) BLV was observed in a study, unlikely to be clinically relevant particularly at standard 2 mg dose. Precautionary clinical monitoring is suggested.
Dofetilide (Antiarrhytmic)	CYP3A4			Weak inhibition of CYP3A4 by high dose (10 mg) BLV was observed in a study, unlikely to be clinically relevant particularly at standard 2 mg dose. Precautionary clinical monitoring is suggested.
Dronedarone (Antiarrhytmic)	CYP3A4			Weak inhibition of CYP3A4 by high dose (10 mg) BLV was observed in a study, unlikely to be clinically relevant particularly at standard 2 mg dose. Precautionary clinical monitoring is suggested.
Quinidine (Antiarrhytmic)	CYP3A4<br>CYP2D6			Weak inhibition of CYP3A4 by high dose (10 mg) BLV was observed in a study, unlikely to be clinically relevant particularly at standard 2 mg dose. Precautionary clinical monitoring is suggested.
Bedaquiline (Antibacterial)	CYP3A4			Weak inhibition of CYP3A4 by high dose (10 mg) BLV was observed in a study, unlikely to be clinically relevant particularly at standard 2 mg dose. Precautionary clinical monitoring is suggested.
Apixaban (Anticoagulant)	P-gp, BCRP<br>CYP3A4			Weak inhibition of CYP3A4 by high dose (10 mg) BLV was observed in a study, unlikely to be clinically relevant particularly at

			standard 2 mg dose. Precautionary clinical monitoring is suggested.
Phenprocoumon (Anticoagulant)	CYP2C9<br>CYP3A4		Weak inhibition of CYP3A4 by high dose (10 mg) BLV was observed in a study, unlikely to be clinically relevant particularly at standard 2 mg dose. Precautionary clinical monitoring is suggested.
Carbamazepine (Anticonvulsant)	CYP3A4<br>CYP2C8		Weak inhibition of CYP3A4 by high dose (10 mg) BLV was observed in a study, unlikely to be clinically relevant particularly at standard 2 mg dose. Precautionary clinical monitoring is suggested.
Tianeptine (Antidepressant)	CYP3A4		Weak inhibition of CYP3A4 by high dose (10 mg) BLV was observed in a study, unlikely to be clinically relevant particularly at standard 2 mg dose. Precautionary clinical monitoring is suggested.
Reboxetine (Antidepressant)	CYP3A4		Weak inhibition of CYP3A4 by high dose (10 mg) BLV was observed in a study, unlikely to be clinically relevant particularly at standard 2 mg dose. Precautionary clinical monitoring is suggested.
Glibenclamide (Antidiabetic)	CYP3A4<br>OATP1B1		Weak inhibition of CYP3A4 by high dose (10 mg) BLV was observed in a study, unlikely to be clinically relevant particularly at standard 2 mg dose. Precautionary clinical monitoring is suggested.
Nateglinide (Antidiabetic)	CYP2C9<br>CYP3A4<br>OATP1B1		Weak inhibition of CYP3A4 by high dose (10 mg) BLV was observed in a study, unlikely to be clinically relevant particularly at standard 2 mg dose. Precautionary clinical monitoring is suggested.<br>BLV inhibited OATPB1/3 in vitro at concentrations that correlate to a high dose (10 mg) in vivo. Coadministration of these substrates should be avoided.
Repaglinide (Antidiabetic)	CYP3A4<br>CYP2C8<br>OATP1B1		Weak inhibition of CYP3A4 by high dose (10 mg) BLV was observed in a study, unlikely to be clinically relevant particularly at standard 2 mg dose. Precautionary clinical monitoring is suggested.<br>BLV inhibited OATPB1/3 in vitro at concentrations that correlate to a high dose (10 mg) in vivo. Coadministration of these substrates should be avoided.
Ketoconazole (Antifungals)		NTCP	Coadministration has not been studied. BLV is catabolized by peptidases and elimination occurs through binding to NTCP. Coadministration with NTCP inhibitors (e.g.ketoconazole) is not recommended as it can alter BLV elimination
Astemizole (Anthistamine)	CYP2D6<br>CYP2J2<br>CYP3A4		Weak inhibition of CYP3A4 by high dose (10 mg) BLV was observed in a study, unlikely to be clinically relevant particularly at standard 2 mg dose. Precautionary clinical monitoring is suggested.
Ebastine (Anthistamine)	CYP3A4		Weak inhibition of CYP3A4 by high dose (10 mg) BLV was observed in a study, unlikely to be clinically relevant particularly at standard 2 mg dose. Precautionary clinical monitoring is suggested.
Fexofenadine (Anthistamine)	P-gp<br>OATP1B1		BLV inhibited OATPB1/3 in vitro at concentrations that correlate to a high dose (10 mg) in vivo. When possible, coadministration of these substrates should be avoided.

Terfenadine (Anthistamine)	CYP3A4		Weak inhibition of CYP3A4 by high dose (10 mg) BLV was observed in a study, unlikely to be clinically relevant particularly at standard 2 mg dose. Precautionary clinical monitoring is suggested.
Dihydroergotamine (Antimigrane agents)	CYP3A4		Weak inhibition of CYP3A4 by high dose (10 mg) BLV was observed in a study, unlikely to be clinically relevant particularly at standard 2 mg dose. Precautionary clinical monitoring is suggested.
Ergotamine (Antimigrane agents)	CYP3A4		Weak inhibition of CYP3A4 by high dose (10 mg) BLV was observed in a study, unlikely to be clinically relevant particularly at standard 2 mg dose. Precautionary clinical monitoring is suggested.
Methylergonovine (Antimigrane agents)	CYP3A4		Weak inhibition of CYP3A4 by high dose (10 mg) BLV was observed in a study, unlikely to be clinically relevant particularly at standard 2 mg dose. Precautionary clinical monitoring is suggested.
Quinine (Antiprotozoals)	CYP3A4		Weak inhibition of CYP3A4 by high dose (10 mg) BLV was observed in a study, unlikely to be clinically relevant particularly at standard 2 mg dose. Precautionary clinical monitoring is suggested.
Aripiprazole (Antipsychotics and neuroleptics)	CYP3A4<br>CYP2D6		Weak inhibition of CYP3A4 by high dose (10 mg) BLV was observed in a study, unlikely to be clinically relevant particularly at standard 2 mg dose. Precautionary clinical monitoring is suggested.
Pimozide (Antipsychotics and neuroleptics)	CYP3A4<br>CYP2D6		Weak inhibition of CYP3A4 by high dose (10 mg) BLV was observed in a study, unlikely to be clinically relevant particularly at standard 2 mg dose. Precautionary clinical monitoring is suggested.
Quetiapine (Antipsychotics and neuroleptics)	CYP3A4		Weak inhibition of CYP3A4 by high dose (10 mg) BLV was observed in a study, unlikely to be clinically relevant particularly at standard 2 mg dose. Precautionary clinical monitoring is suggested.
Nirmatrelvir/Ritonavir (Paxlovid) (Antivirals)		NTCP (ritonavir)	BLV is catabolized by peptidases and elimination occurs through binding to NTCP. Coadministration with NTCP inhibitors (e.g.ritonavir) is not recommended as it may alter BLV elimination. Given the short duration of nirmatrelvir/ritonavir therapy for COVID-19, consider pausing BLV and restarting 3 days after completing nirmatrelvir/ritonavir treatment.
Brincidofovir (Antivirals)	NTCP<br>OATP1B1		As a precautionary measure, close clinical monitoring is warranted when NTCP and OATP1B1 substrates (e.g.brincidofovir) are coadministered with BLV. Where possible, coadministration of these substrates should be avoided. If concomitant use with brincidofovir is necessary, increase the monitoring for adverse reactions associated with brincidofovir (i.e.elevations in transaminases and bilirubin, diarrhea, orothergastro-intestinal adverse events) and postpone the dosing of BLV for at least 3 hours after brincidofovir administration.
Midazolam (Anxiolitics, sedatives and hypnotics)	CYP3A4		In a clinical study, an approximately 40% increase in AUC values of coadministered midazolam (CYP3A4 substrate) was observed in combination with high dose BLV (10 mg). Caution is recommended when coadministering.

Everolimus (Cancer Therapies)	CYP3A4<br>P-gp	Weak inhibition of CYP3A4 by high dose (10 mg) BLV was observed in a study, unlikely to be clinically relevant particularly at standard 2 mg dose. Precautionary clinical monitoring is suggested.
Paclitaxel (Cancer Therapies)	CYP2C8<br>CYP3A4<br>OATP1B1	BLV inhibited OATPB1/3 in vitro at concentrations that correlate to a high dose (10 mg) in vivo. When possible, coadministration of these substrates should be avoided.
Sunitinib (Cancer Therapies)	CYP3A4	Weak inhibition of CYP3A4 by high dose (10 mg) BLV was observed in a study, unlikely to be clinically relevant particularly at standard 2 mg dose. Precautionary clinical monitoring is suggested.
Temsirolimus (Cancer Therapies)	CYP3A4	Weak inhibition of CYP3A4 by high dose (10 mg) BLV was observed in a study, unlikely to be clinically relevant particularly at standard 2 mg dose. Precautionary clinical monitoring is suggested.
Trastuzumab emtansine (Cancer Therapies)	DM-1 CYP3A4<br>CYP3A5	Weak inhibition of CYP3A4 by high dose (10 mg) BLV was observed in a study, unlikely to be clinically relevant particularly at standard 2 mg dose. Precautionary clinical monitoring is suggested.
Olaparib (Cancer Therapies)	CYP3A4/5	Weak inhibition of CYP3A4 by high dose (10 mg) BLV was observed in a study, unlikely to be clinically relevant particularly at standard 2 mg dose. Precautionary clinical monitoring is suggested.
Ibrutinib (Cancer Therapies)	CYP3A4<br>CYP2D6	A small increase in ibrutinib concentrations may occur. No a priori dose adjustment is required, but monitor patient closely for toxicity and refer to product label for dose adjustment if clinically relevant.
Idelalisib (Cancer Therapies)	Aldheyde oxidase<br>CYP3A4<br>UGT1A4	Weak inhibition of CYP3A4 by high dose (10 mg) BLV was observed in a study, unlikely to be clinically relevant particularly at standard 2 mg dose. Precautionary clinical monitoring is suggested.
Panobinostat (Cancer Therapies)	Non-CYP and CYP mediated routes.<br>~ 40% of panobinostat is metabolised by CYP3A4	Weak inhibition of CYP3A4 by high dose (10 mg) BLV was observed in a study, unlikely to be clinically relevant particularly at standard 2 mg dose. Precautionary clinical monitoring is suggested.
Ergometrine (Oxytocics)	CYP3A4	Weak inhibition of CYP3A4 by high dose (10 mg) BLV was observed in a study, unlikely to be clinically relevant particularly at standard 2 mg dose. Precautionary clinical monitoring is suggested.
Colchicine	CYP3A4<br>Pgp	Weak inhibition of CYP3A4 by high dose (10 mg) BLV was observed in a study, unlikely to be clinically relevant particularly at standard 2 mg dose. Precautionary clinical monitoring is suggested.
Levothyroxine	NTCP	As a precautionary measure, close clinical monitoring is warranted when NTCP substrates are coadministered with BLV. Where possible, coadministration of these substrates should be avoided.

Ezetimibe (Lipid Lowering Agents)			NTCP	Coadministration has not been studied. BLV is catabolized by peptidases and elimination occurs through binding to NTCP. Co-administration with NTCP inhibitors is not recommended as it can alter BLV elimination
Atorvastatin (Lipid Lowering Agents)	NTCP OATP1B1			BLV inhibited OATPB1/3 in vitro at concentrations that correlate to a high dose (10 mg) in vivo. When possible, coadministration of these substrates should be avoided.
Fluvastatin (Lipid Lowering Agents)	NTCP			As a precautionary measure, close clinical monitoring is warranted when NTCP substrates (e.g.fluvastatin) are coadministered with BLV. When possible, coadministration of these substrates should be avoided.
Lovastatin (Lipid Lowering Agents)	CYP3A4 OATP1B1			BLV inhibited OATPB1/3 in vitro at concentrations that correlate to a high dose (10 mg) in vivo. As a precautionary measure, close clinical monitoring is suggested for coadministered narrow-therapeutic index drugs which are sensitive OATP1B1 substrates.
Pitavastatin (Lipid Lowering Agents)	NTCP OATP1B1			BLV inhibited OATPB1/3 in vitro at concentrations that correlate to a high dose (10 mg) in vivo. When possible, coadministration of these substrates should be avoided.
Pravastatin (Lipid Lowering Agents)	NTCP OATP1B1			BLV inhibited OATPB1/3 in vitro at concentrations that correlate to a high dose (10 mg) in vivo. When possible, coadministration of these substrates should be avoided.
Rosuvastatin (Lipid Lowering Agents)	NTCP OATP1B1			BLV inhibited OATPB1/3 in vitro at concentrations that correlate to a high dose (10 mg) in vivo. When possible, coadministration of these substrates should be avoided.
Simvastatin (Lipid Lowering Agents)	CYP3A4 BCRP OATP1B1			Weak inhibition of CYP3A4 by high dose (10 mg) BLV was observed in a study, unlikely to be clinically relevant particularly at standard 2 mg dose. BLV inhibited OATPB1/3 in vitro at concentrations that correlate to a high dose (10 mg) in vivo. Precautionary clinical monitoring is suggested.
Cyclosporin (Immunosuppressants)			NTCP	Coadministration has not been studied. BLV is catabolized by peptidases and elimination occurs through binding to NTCP. Coadministration with NTCP inhibitors is not recommended as it can alter BLV elimination
Sirolimus (Immunosuppressants)	CYP3A4			Weak inhibition of CYP3A4 by high dose (10 mg) BLV was observed in a study, unlikely to be clinically relevant particularly at standard 2 mg dose. Precautionary clinical monitoring is suggested.
Tacrolimus (Immunosuppressants)	CYP3A4			Weak inhibition of CYP3A4 by high dose (10 mg) BLV was observed in a study, unlikely to be clinically relevant particularly at standard 2 mg dose. Precautionary clinical monitoring is suggested.
Irbesartan (Hypertension, Heart Failure agents)			NTCP	Coadministration has not been studied. BLV is catabolized by peptidases and elimination occurs through binding to NTCP. Coadministration with NTCP inhibitors (e.g.irbesartan) is not recommended as it can alter BLV elimination
Ambrisentan (Hypertension, Heart Failure agents)	CYP3A4 P-gp OATP1B1			BLV inhibited OATPB1/3 in vitro at concentrations that correlate to a high dose (10 mg) in vivo. Precautionary clinical monitoring is suggested.
Bosentan	CYP3A4 CYP2C9 OATP1B1/3	weak inducer of CYP3A4		BLV inhibited OATPB1/3 in vitro at concentrations that correlate to a high dose (10 mg) in vivo. Precautionary clinical monitoring is suggested.

(Hypertension, Heart Failure agents)		and CYP2C9		
Olmesartan (Hypertension, Heart Failure agents)	OATP1B1			BLV inhibited OATPB1/3 in vitro at concentrations that correlate to a high dose (10 mg) in vivo. When possible, coadministration of these substrates should be avoided.
Telmisartan (Hypertension, Heart Failure agents)	OATP1B1			BLV inhibited OATPB1/3 in vitro at concentrations that correlate to a high dose (10 mg) in vivo. Precautionary clinical monitoring is suggested. Considering the good tolerability of telmisartan, no clinically relevant DDI is expected.
Sacubi-tril/Valsartan (Hypertension, Heart Failure agents)	OATP1B1/3			BLV inhibited OATPB1/3 in vitro at concentrations that correlate to a high dose (10 mg) in vivo. Precautionary clinical monitoring is suggested. When possible, coadministration of these substrates should be avoided.
Sulfasalazine (Gastrointestinal Agents)			NTCP	Coadministration has not been studied. BLV is catabolized by peptidases and elimination occurs through binding to NTCP. Co-administration with NTCP inhibitors is not recommended as it can alter BLV elimination
Cisapride (Gastrointestinal Agents)	CYP3A4			Weak inhibition of CYP3A4 by high dose (10 mg) BLV was observed in a study, unlikely to be clinically relevant particularly at standard 2 mg dose. Precautionary clinical monitoring is suggested.
Domperidone (Gastrointestinal Agents)	CYP3A4			Weak inhibition of CYP3A4 by high dose (10 mg) BLV was observed in a study, unlikely to be clinically relevant particularly at standard 2 mg dose. Precautionary clinical monitoring is suggested.

Hypothesised BLV interactions with compound belonging to other drug classes. All the information reported in this table were obtained from the HEP Drug Interactions database (University of Liverpool https://hep-druginteractions.org/checker, accessed on 16 Dec. 2024).
